# Supplementary figures and images for: Mitochondrial Genome of Fagus sylvatica L. as a Source for Taxonomic Marker Development in the Fagales
Source: Plants (Basel). 2020 Sep 27;9(10):1274. doi: 10.3390/plants9101274 (PMC7650814; doi:10.3390/plants9101274)

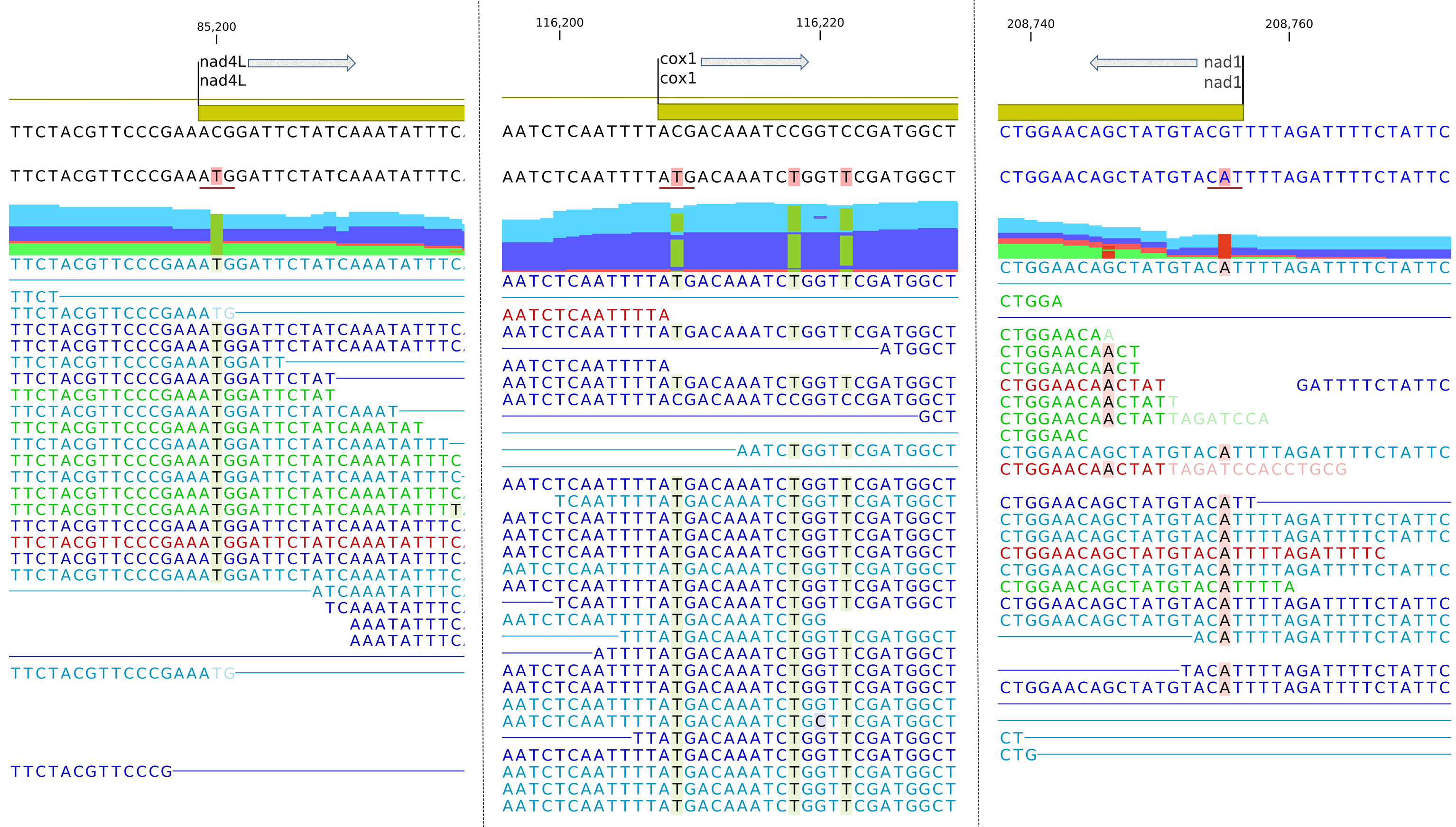

Supplement: Supplementary file 1 [file plants-09-01274-s001.zip › Supplementals/Figure_S2.tif]

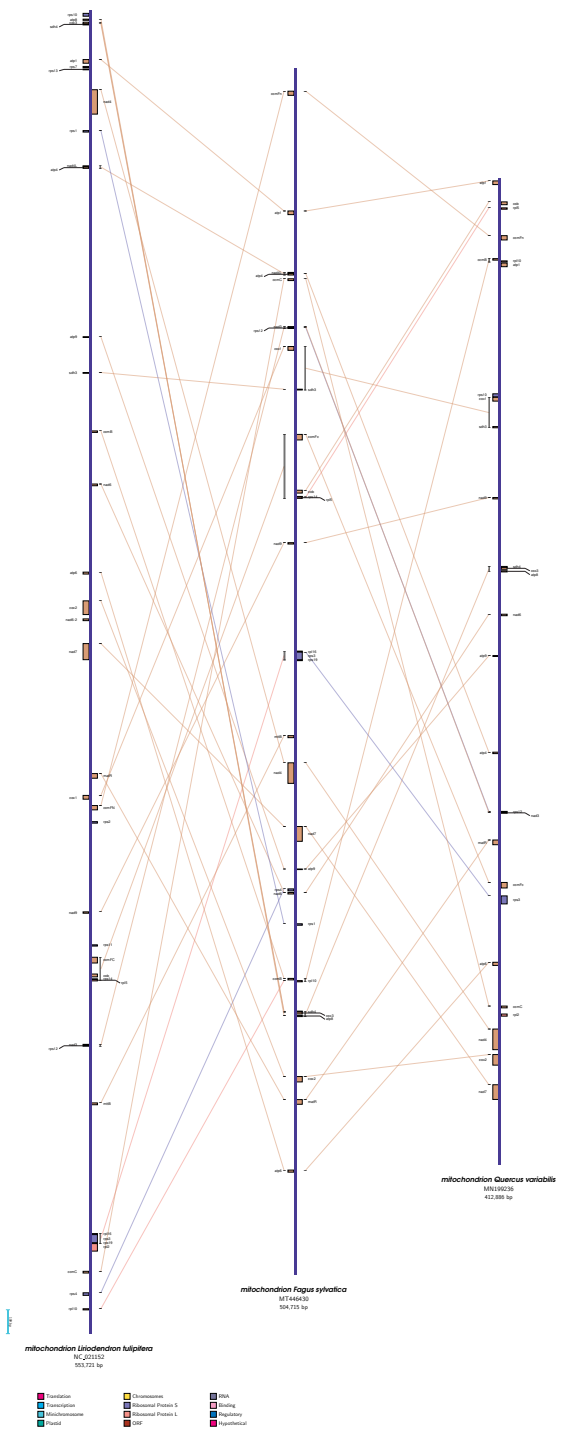

Supplement: Supplementary file 1 [file plants-09-01274-s001.zip › Supplementals/Figure_S3.pdf]

*Fagus  
sylvatica*

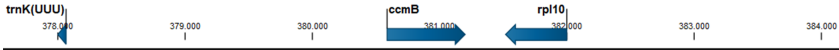

*Quercus  
variabilis*

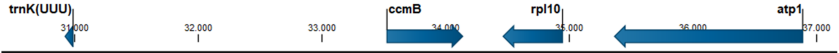

*\*Betula  
pendula*

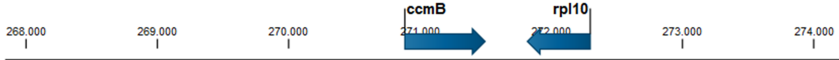

Supplement: Supplementary file 1 [file plants-09-01274-s001.zip › Supplementals/Figure_S4.pdf]
